# Polylactic Acid (PLA) Biocomposite: Processing, Additive Manufacturing and Advanced Applications

**DOI:** 10.3390/polym13081326

**Published:** 2021-04-18

**Authors:** R.A. Ilyas, S.M. Sapuan, M.M. Harussani, M.Y.A.Y. Hakimi, M.Z.M. Haziq, M.S.N. Atikah, M.R.M. Asyraf, M.R. Ishak, M.R. Razman, N.M. Nurazzi, M.N.F. Norrrahim, Hairul Abral, Mochamad Asrofi

**Affiliations:** 1 School of Chemical and Energy Engineering, Faculty of Engineering, Universiti Teknologi Malaysia, UTM Johor Bahru 81310, Johor, Malaysia; 2 Centre for Advanced Composite Materials, Universiti Teknologi Malaysia, UTM Johor Bahru 81310, Johor, Malaysia; 3 Laboratory of Biocomposite Technology, Institute of Tropical Forestry and Forest Products (INTROP), Universiti Putra Malaysia, UPM Serdang 43400, Selangor, Malaysia; 4 Advanced Engineering Materials and Composites (AEMC), Department of Mechanical and Manufacturing Engineering, Faculty of Engineering, Universiti Putra Malaysia, UPM Serdang 43400, Selangor, Malaysia; mmharussani17@gmail.com (M.M.H.); yuzihakimi23@gmail.com (M.Y.A.Y.H.); haziqz.dev@gmail.com (M.Z.M.H.); 5 Department of Chemical and Environmental Engineering, Universiti Putra Malaysia, UPM Serdang 43400, Selangor, Malaysia; sitinuratikah_asper7@yahoo.com; 6 Department of Aerospace Engineering, Faculty of Engineering, Universiti Putra Malaysia, UPM Serdang 43400, Selangor, Malaysia; asyrafriz96@gmail.com (M.R.M.A.); mohdridzwan@upm.edu.my (M.R.I.); 7 Research Centre for Sustainability Science and Governance (SGK), Institute for Environment and Development (LESTARI), Universiti Kebangsaan Malaysia, UKM Bangi 43600, Selangor, Malaysia; 8 Centre for Defence Foundation Studies, Universiti Pertahanan Nasional Malaysia (UPNM), Kem Perdana Sungai Besi 57000, Kuala Lumpur, Malaysia; mohd.nurazzi@gmail.com; 9 Research Center for Chemical Defence, Universiti Pertahanan Nasional Malaysia (UPNM), Kem Perdana Sungai Besi 57000, Kuala Lumpur, Malaysia; faiznorrrahim@gmail.com; 10 Department of Mechanical Engineering, Andalas University, Padang 25163, Sumatera Barat, Indonesia; habral@yahoo.com; 11 Department of Mechanical Engineering, University of Jember, Kampus Tegalboto, Jember 68121, East Java, Indonesia; asrofi.teknik@unej.ac.id

**Keywords:** polylactic acid (PLA), natural fibres, biocomposite, mechanical properties

## Abstract

Over recent years, enthusiasm towards the manufacturing of biopolymers has attracted considerable attention due to the rising concern about depleting resources and worsening pollution. Among the biopolymers available in the world, polylactic acid (PLA) is one of the highest biopolymers produced globally and thus, making it suitable for product commercialisation. Therefore, the effectiveness of natural fibre reinforced PLA composite as an alternative material to substitute the non-renewable petroleum-based materials has been examined by researchers. The type of fibre used in fibre/matrix adhesion is very important because it influences the biocomposites’ mechanical properties. Besides that, an outline of the present circumstance of natural fibre-reinforced PLA 3D printing, as well as its functions in 4D printing for applications of stimuli-responsive polymers were also discussed. This research paper aims to present the development and conducted studies on PLA-based natural fibre bio-composites over the last decade. This work reviews recent PLA-derived bio-composite research related to PLA synthesis and biodegradation, its properties, processes, challenges and prospects.

## 1. Introduction

Since the early 1970s, biodegradable polymers have been attracting the focus of researchers on their development as a result of increasing concern about material resources as well as plastic disposal issues [1,2,3,4]. Composite materials are formed within the complete structure of more than one material. The bulk of composites have solid and rigid fibres with a low density in a matrix. Polylactic acid (PLA) is a flexible polymer that is fermented into a carboxylic acid and made from sustainable agricultural waste [5,6]. The lactic acid is then polymerized through a cyclic dilactone, lactide, and ring for product modification. The growing awareness of environmental sustainability and new laws and policies has pushed companies to produce environmentally friendly products [7].

A lot of studies are performed that aim to produce entirely biodegradable composite structures with PLA and natural fibres combination [8]. Since both PLA and natural fibres are sourced from renewable sources, biodegradable, as well as biocompostable, natural/PLA composites are recyclable green materials. Thus, the biocomposites have significant advantages due to their reduced manufacturing and waste disposal treatment costs, as natural fibre reinforced composites can be disposed of easily via landfill, incineration, or by green treatment of pyrolysis, as stated by Harussani et al. [2]. In addition, biopolymers are suitably utilised in various methods of composite fabrication, for example, injection moulding, extrusion, compression moulding, and so forth, whereas less research had been conducted on composites derived from recycled raw materials with matrixes. Biopolymers also satisfy the long-term characteristics of sustainable materials because they are not single-use products. The investigation by Oksman et al. [9] showed that the strength indicated by the stiffness of glass fibre composites was lower than the natural fibre composites. Moreover, the adhesion of the fibre/matrix is a dynamic process with several overlapping variables. Graupner [10] believed that lignin has the ability to strengthen the fibre and matrix bond that is proven by the production of new advanced types of natural fibre reinforced PLA composite.

The reinforcement of PLA composite with natural fibre is a key factor in increasing biocomposites applications in the mechanical field. In addition, among biodegradable polymers, bio polyester PLA is recorded with the highest volume applications for industrial demands. This is attributed to the effective life cycle assessment of PLA, where the supply chain of PLA requires less transportation as well as emits fewer greenhouse gases. The significant properties, such as biodegradability, renewability and lower CO_2_ emissions, of PLA-based biocomposites led to their performance in the market. Thus, this review aims to investigate the inherent properties of PLA itself and natural fibre, as well as their synergistic effects when hybridized together. This paper comprises a compilation of previous works and the development of PLA/natural fibre composites in industrial fields.

## 2. Natural Fibre

**Figure 1:**
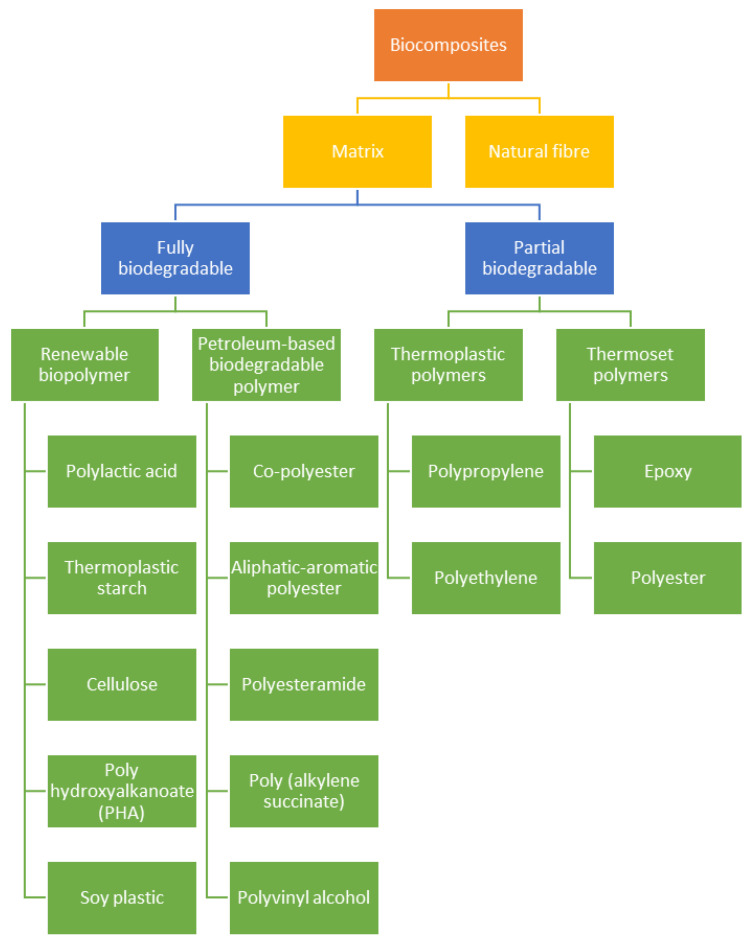
Classification of biocomposites. Adapted with copyright permission from Mohanty et al. [67].

**Figure 2:**
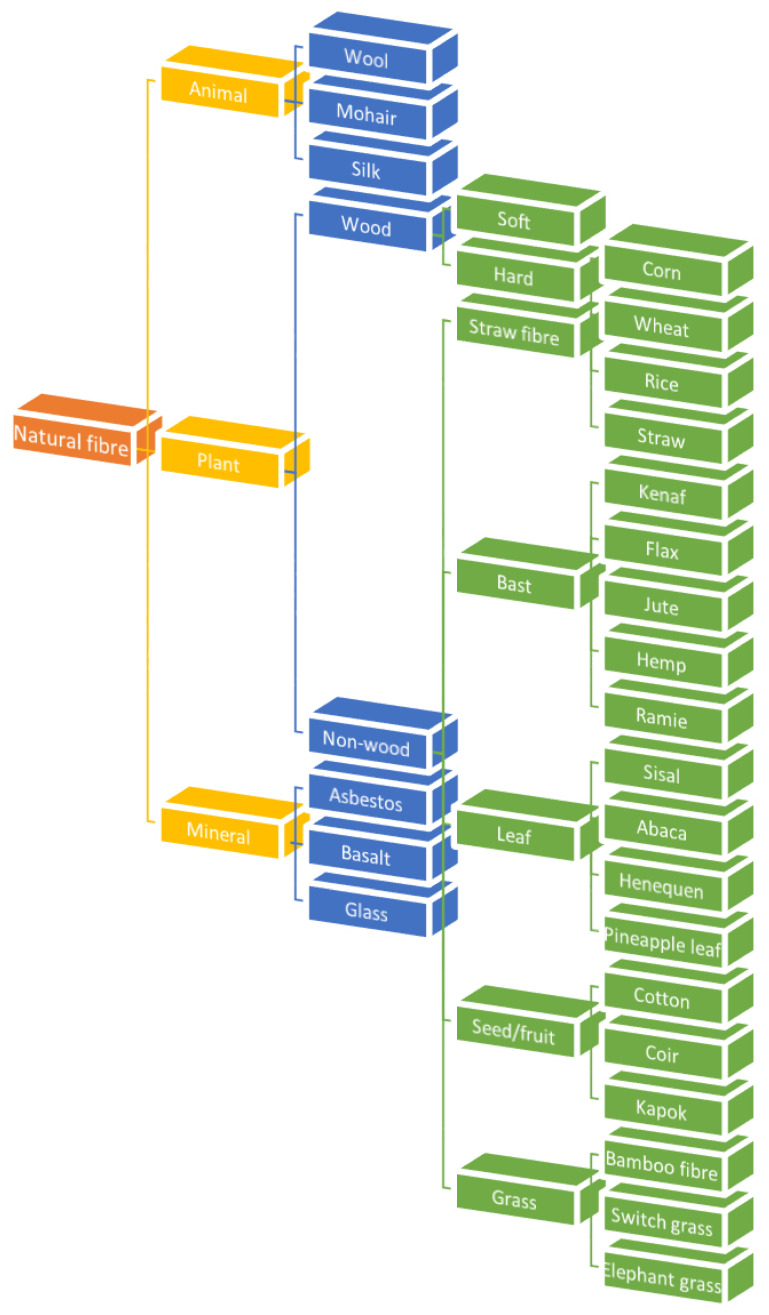
Classification of natural fibres.

Natural fibre can be obtained from plants, animals or the environment. Figure 1 and Figure 2 show basic information about the branches of biocomposites and natural fibres classification. The advantages of natural fibres over synthetic fibres are their recyclability and biodegradability [11,12,13]. The natural fibre market and production are drastically progressing, shifting the focus of science and engineering to PLA composites. Nowadays, natural fibres are well known in reinforced polymeric materials for industrial developments, for example, glass fibre as a matrix material [14,15,16,17]. The polymer is obtained from the fermentation of corn, potato, sugar, beet, and other agricultural sources. Despite their biodegradability properties, natural fibres exhibit several main drawbacks that hinder their developments, including differences in consistency, sensitivity to moisture intake due to their hydrophilic nature, and low thermal stability [18,19,20,21].

Studies on the processing effects and natural fibres properties show an improvement in mechanical properties parallel to the National Policy on Industry 4.0 (Industry 4WRD). Natural fibres have sparked great interest among researchers and industry players for their applications in the military [22], automotive [23,24,25,26,27], industrial [28,29,30,31,32,33], furniture [34], civil [35,36], and biomedical fields [37]. It is reported that the natural fibres global production exceeded 105 million metric tons in 2018 [38]. Mignaeault et al. [39] studied the applications of wood-plastic composites that are typically manufactured as hybrid products by combining wood flour with recycled plastic. The studies on the fibre length and processing effects on the composite’s properties revealed enhanced mechanical properties with increasing fibre content. The non-wood fibres include plant straw, leaf, bast, fruit, seed, or grass. Straw fibres originating from resources such as maize, wheat, and rice hull are comparatively solid, rigid, low density, and sustainable. These natural fibre reinforced composites are used as deck boards in housing construction materials [40].

Biopolymer PLA has long been a matter of considerable concern in multiple sectors. This is because, in fibre or matrix adhesion, the type of fibre plays a significant role and that influences the biocomposite’s mechanical performance. Recent evidence by Mukherjee and Kao [40] showed that the cellulose content and the microfibrils angle are the two variables that govern the mechanical properties of natural fibre. The better the mechanical properties of the fibre, the better the mechanical properties of the biocomposites. The mechanical properties of natural fibres depend on the cellulose content in them. Table 1 shows the chemical composition of the natural fibres, where the natural fibres’ chemical composition and cell structures are quite complex and differ in plant parts and origins. Depending on the cellulose crystallinity, the physical, chemical, and mechanical behaviours of the lignocellulosic fibres vary from one another [41]. Type of fibre is an important parameter in determining their chemical composition. The primary natural fibres components comprise cellulose, hemicellulose and lignin [42,43,44]. Each fibre exists as a composite by nature, where the rigid cellulose microfibrils are protected in the amorphous matrix containing hemicellulose and lignin [45,46,47,48,49]. Thus, natural fibres can also be identified as cellulosic or lignocellulosic fibres [43]. Generally, the main natural fibres’ constituent is cellulose with 30%–80% followed by hemicellulose of 7%–40%, and 3%–33% lignin as shown in Table 1.

Besides this, the surface structure of the natural fibre plays a vital role in the adhesion behaviour of the matrix polymer and the fibres. The higher and stronger the fibres and matrix adhesion, the better the natural fibres’ mechanical properties. The composite intensity depends on the various level of the fibres in use and is approximately equal to its matrix binding mechanism.

### Chemical Treatment of Natural Fibres

Various studies have assessed the efficacy of reinforced natural fibre polymer composite in a variety of fields. In recent years, a number of treatments were developed to enhance interfacial bonding and to improve mechanical properties and water-resistance characteristics of natural fibres. The most critical downside of these natural fibres in polymer composites is their hydrophilicity that creates a weak interfacial bonding between fibres and matrix. Numerous physical impurities and hydroxyl groups are found on the surface of the fibres that affect their performance as reinforcing agents. Thus, several surface modifications and treatments have been explored in order to obtain the desired properties of polymer composites.

To identify a knowledge gap in the field of study in natural fibres reinforced PLA composites, a review by Sawpan et al. [68] on the improvement of mechanical performance of industrial hemp fibre reinforced polylactide biocomposites was conducted. Recently, simpler and more rapid tests of PLA have been developed for a variety of fibre material properties of random fibres. Interestingly, aligned PLA composites reinforced with long hemp fibre (0–40 wt.%) were studied. It was revealed that 30% alkali-treated fibre reinforced PLA composite (PLA/ALK) had the highest mechanical strength with a tensile strength of more than 70 MPa, a Young’s modulus of more than 8 GPa module, and flexural toughness of 2.64 kJ/m^2^. The use of qualitative case studies is a well-established approach in the alignment of long fibres along with the improvement of PLA/ALK composites.

A previous literature review by Li et al. [69] focused on the effect of chemical treatments on natural fibres for the application of natural fibre-reinforced composites. The authors identified poor fibre-matrix compatibility and relatively high absorption of moisture as the key drawbacks of natural fibres in composites. Thus, modifying the fibre surface properties using chemical treatments were being contemplated. Chemical treatments, for example, alkali, silane, acetylation, benzoylation, and so forth were found to improve fibre-matrix surface adhesion and reduce the water absorption properties of the fibres [19]. In order to facilitate the adhesion, chemical coupling of the adhesive with certain compounds, for example, sodium hydroxide, acetic acid, silane, acrylic acid, isocyanates, maleate coupling agents, potassium permanganate and peroxide was applied. Data from this study showed that chemical treatments should be applied when modifying the properties of natural fibres.

Reinforcing natural fibre with polymer composite has been a major focus of previous research into the natural fibre. In a study on natural fibre surface modifications and performance of the subsequent bio-composites, Mohanty et al. [67] stated that an adequate degree of adhesion between the surface of hydrophilic natural fibre cellulose and the polymer matrix resin is usually needed to ensure the desirable properties of bio-composites. Useful methods to enhance fibre-matrix adhesion in natural fibre composites are alkali, peroxide, coupling agents and isocyanate treatments, dewaxing, bleaching, vinyl grafting and acetylation. By replacing the hydroxyl group with some chemical functionality, the crystalline structure of cellulose can be broken. This process of decrystallization enhances the thermoplasticity of the cellulose because the role of the plasticiser is performed by the substituted groups [70]. The findings of this study suggested that a few levels of success in creating a superior interface were achieved by surface modifications of hydrophilic natural fibres, but cheaper cost surface modification needs to be emphasised in many applications in the future for biocomposites to replace glass fibre composites.

## 3. Polylactic Acid (PLA)

**Figure 3:**
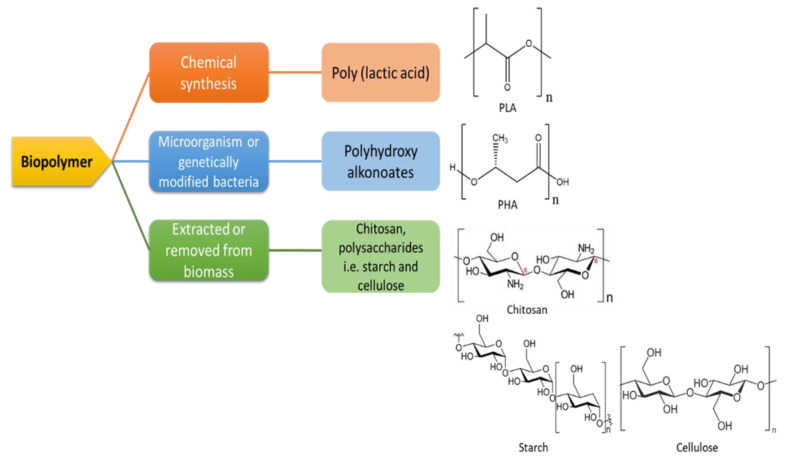
Chemical structure and classification of significant biopolymers based on their origin. Reproduced with copyright permission from Mohanty et al. [81].

Poly (lactic acid) (PLA) exits in the form of polymers that are biodegradable polyesters obtained from lactic acid (LA) or 2-hydroxy propionic acid, typically obtained from agricultural crops such as maize, potatoes, and cassava through bacterial fermentation of carbohydrates. As shown in Figure 3, PLA is categorised as a biopolymer originated from chemically treated bio-based monomers, LA [71]. Owing to its biocompatibility with the human body, PLA has been the choice of material for medical applications [72]. The discovery of the latest polymerisation pathways that enable high molecular weight PLA to be produced economically, together with the rise of awareness on environmental issues have led to the broadening of the use of PLA as alternative raw materials [73].

PLA is the most widely researched and promising biopolymer that is capable of substituting conventional petroleum-based polymers due to its renewability, recyclability, biodegradability and compostability. In addition, PLA has an excellent manufacturing ability as it is suitable to be processed with various methods. Injection moulding, film extrusion, blow moulding, thermoforming, fibre spinning and film-forming are among the PLA manufacturing processes [74]. PLA-derived products had utilized in several industrial applications, including in packaging [8,75], textile [76], biomedical [77], structural [78,79] and automotive [80].

To determine the properties of PLA, Sodergard et al. [72] stated that PLA polymers are obtained from agricultural crops such as maize and potatoes through bacterial fermentation of carbohydrates. Three synthesis processes of PLA were used by Sodergard et al. [72] to collect data. Then, there are a lot of advantages of using PLA as composites, for example, minimized weight, decreased thermal conductivity and suitable for medical implant applications as identified in this study. PLA with greater mechanical and flexural characteristics has improved the temperature of heat distortion and improved membrane characteristics.

## 4. Composite

Composite materials could be described as materials that consist of two or more phases separated by different interfaces that are chemically and physically dissimilar [82,83,84].

**Figure 4:**
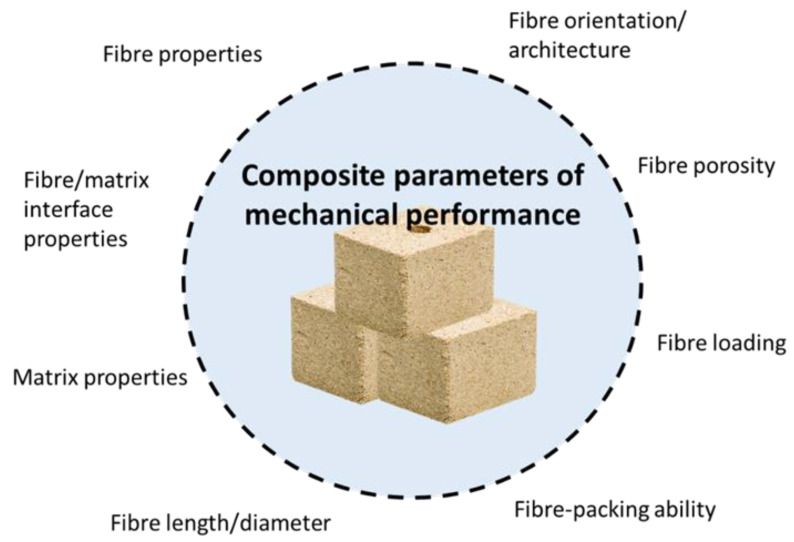
Mechanical properties of the composite.

The various systems are carefully integrated in order to attain a system with more valuable structural or functional properties which cannot be obtained by either of the components alone [85,86,87]. In general, composite materials exhibit special features in which the strength-to-weight ratio is high. One other benefit of composite material is that it offers structural versatility since it is possible to form composites into complex shapes [88]. Composite materials obtain the majority of their advantageous properties through a tight bond between a strong rigid reinforcement—commonly fibres (filaments) or reinforcements with other geometric forms, such as particles, platelets—and a weaker, less rigid matrix [89]. The basic composite components and features giving the mechanical properties of the composite are shown in Figure 4.

A number of studies have begun to examine natural fibres reinforced PLA composites. The analysis was based on the factors influencing the performance of tensile strength. Based on the article written by Ku et al. [90], the composites’ tensile strength with fibres was found to be 20–40% lower in the perpendicular direction than that of composites with fibres in the parallel direction. The mean score for natural fibre was it might be used to substitute traditional fibres in plastic content reinforcement. For natural fibre reinforcements, matrix interfacial bonding, new manufacturing methods and chemical and physical alteration techniques are developed, and the tensile properties of the composites are improved.

## 5. Natural Fibre Reinforced PLA Composite

### 5.1. Brief on PLA Composite

**Figure 5:**
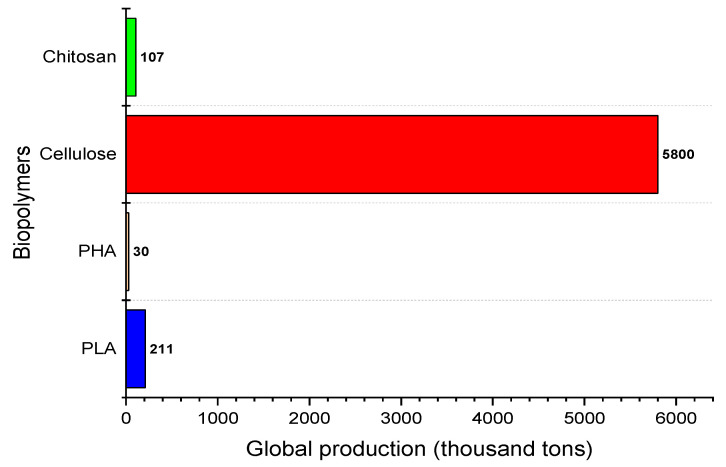
Global production of biopolymers in the year 2020 [91,92,93].

PLA is a naturally resourced thermoplastic polymer produced globally with a capacity of approximately 211,000 tons in 2020 [91]. In addition, chitosan and chitin derivatives productions were about 107,000 tons [92], while cellulose and PHA were produced globally with a capacity of over 580,000 tons and 30,000 tons, respectively, in 2020 [93]. These data had been represented as graph bar in Figure 5. The global market for PLA is estimated to double every four years according to Jem’s law [91]. This is supported by the fact that environmental pollution due to excessive petroleum-derived plastic production and global warming pressure had attracted the public’s attention to extensively explore the utilisation of biodegradable products.

According to Nazrin et al. [8], biodegradable polymers PLA are brittle, sensitive to moisture and have low-impact strength. Thus, a possible way to further reinforce the polymers is by hybridising them with natural fibres to yield the enhanced mechanical properties of biocomposite. Moreover, biocomposites are biodegradable, reusable, and organic-based, assisting to reduce both the reliance on depleting petroleum sources and the environmental burdens from petroleum-based products. The PLA’s excellent mechanical properties and barrier capability can be used to manufacture biomaterials that suit a range of applications.

A variety of studies have begun to investigate PLA-based composite with natural fibres reinforcement. A recent systematic literature review on natural fibre-reinforced PLA composites by Siakeng et al. [94] successfully demonstrated the developments in the study of natural fibre composites based on PLA and neat PLA over the past couple of years. A comprehensive investigation was conducted to understand the characteristics of natural fibre and PLA and their activities after a variety of processing techniques. From the study, it was shown that natural fibre reinforced PLA composite can provide a possible alternative to replace the utilisation of synthetic polymers that are unsustainable and hard to dispose. However, there has been limited progress in attempting to build entirely biodegradable composites from biopolymers. This study points out the efficient replacement of synthetic polymer composites with fully biodegradable composites would need an improvement in terms of PLA manufacturing cost.

Hinchcliffe et al. [95] integrated the theoretical and experimental researches on prestressed natural fibre reinforced PLA composite components. In the study, the mechanical properties of PLA were enhanced with additive loading. Using both tensile and flexural mechanical tests, the effect of fibre form, matrix cross-section geometry PLA, and post-tension level of the PLA were mechanically investigated. In contrast to solid non-reinforced PLA composites, the PLA reinforced composite showed increment in tensile strength by 116% and flexural specific strength by 12%. This study revealed the possibility of basic tensile and flexural strengths’ improvements of PLA composites by creating increasingly efficient structural shapes using additive manufacturing (3D printing) and by processes such as initial post-tensioning fibre.

Many researchers have utilised natural fibres reinforced polylactide (PLA) composites. Several developments focused on PLA due to properties greater than conventional polymers. The mean score for natural fibres was most of the layer and mass modification techniques were optimized to change a specific property and had also ignored the effect of the technique on other primary properties. The effectiveness of bulk and surface modification methods of widely used thermoplastic polyesters on 3-dimensional (3D) scaffolds (PHA) is reported in [74]. The findings will be of interest to investigate the work of PLA shape memory properties.

### 5.2. Development of Natural Fibres/PLA Biocomposite

#### 5.2.1. Brief Study on PLA Biocomposite

A lot of studies have investigated natural fibres reinforced polylactide (PLA) composites in any systematic way. There is a growing body of literature that recognises the importance of combination biodegradable polymers with plant fibres to produce composites. Table 2 summarises works on natural fibre reinforced PLA composites. A seminal study from Holbery and Houston [96] carried out the application of natural fibre reinforced polymer composites in the automotive industry. Natural fibres, for example, kenaf, hemp, flax, jute and sisal were found to offer benefits such as weight, cost, CO_2_ reduction, less dependency on external oil sources and recyclability. The production of the composite involved the combination of natural fibre preform or mats along with a thermoplastic binder system. The most popular system currently used in vehicle industries is thermoplastic polypropylene, especially for non-structural components. In a variety of automotive components, using natural fibre reinforcement is proven feasible. The applications of seatback linings, door cladding, and floor panels are refined from flax, sisal, and hemp. Coconut fibre is used in the manufacturing of seat bottoms, head restraints, and back cushions, while cotton can be used for sound barrier and wood fibre is often used in the backseat. Only if problematic areas such as moisture issues, compatibility, durability, and continuous fibre source could be tackled by vehicle manufacturers, can natural fibre composites compete as reliable materials in the automotive industry.

In a review conducted by Dunne et al. [97], the sustainability and automotive applications of natural fibres were compiled. Based on previous research, there is a lot of new advancement for humanity in the production of natural fibres for industrial use. This trend is attributed not only to an increased awareness of the environment but also to the fact that natural fibres have excellent properties. At the moment, there is still a lot of ongoing research on natural fibres and their ability to replace petroleum-based products, and only time will tell which of the two will be the better option. Faruk et al. [98] developed a study on biocomposites reinforced with natural fibres. The physical, thermal and mechanical properties of the biocomposites could be affected by various factors, including the type of fibre, conditions for the climate, manufacturing processes and surface modification of the fibre. A broad range of biocomposite processing techniques and variables (moisture quality, form and content of fibre, binding agents and their effect on the properties of composites) influence these processes.

Results from several studies conducted by Herrera-Franco and Valadez-Gonzalez [99] about mechanical properties of continuous natural fibre-reinforced polymer composites showed positive results. Strong evidence of natural fibres reinforced PLA composites was found when ASTM D 638-99 was tested using an Instron 6025 100 kN test machine. Fibres samples analysed (10% of waste fibres) using the silane concentration struggled in a brittle manner to facilitate a chemical interrelationship and fibre-matrix binding. The result took three weeks for the equilibrium conditioning at 65% relative humidity and final moisture. Whereas, for transverse tensile and flexural properties, the rise was greater than 50% corresponding to the untreated fibre composite direction. The findings of this analysis showed that it was found from the failure surfaces that the fibre matrix enhanced the association of the failure mode.

The topic of natural fibre reinforced PLA composites was investigated a long time ago. Research by Alkbir et al. [100] was carried out about natural fibre-reinforced composite structure fibre characteristics and chassis rigidity parameters. From the findings, materials, for example, jute, sisal, flax, kenaf and hemp can be used in various industrial applications due to their outstanding mechanical features, low cost, high strength, eco-friendliness and biodegradability, ease of processing and strong rigidity of the structure. The total tensile strength and bending strength characteristics of natural fibre-reinforced polymer hybrids were strongly focused on the ratio of water uptake. The findings will be of interest to geometric structures between two things that were found to have a major effect on the parameters of crashworthiness and basic load transfer of polymer composite tubes reinforced with natural fibre.

#### 5.2.2. Non-Wood Natural Fibre/PLA Composite

##### Leaf

The efficacy of natural fibre reinforcements with PLA composite was tested in various studies. To date, several studies were carried out on strengthening the PLA-based composites using leaf originated natural fibres including sisal and leaf fibre. From previous research by Asaithambi et al. [101] on reinforced PLA hybrid composites with banana/sisal fibre (BSF), the impact of benzoyl peroxide (BP) surface treatment on the mechanical properties of BSF-reinforced PLA composites was investigated. BSF reinforced PLA hybrid composites were manufactured using the twin-screw extrusion method followed by injection moulding. Using the Universal Testing Machine (UTM), tensile strength and flexural strength were evaluated. The results of the study suggested that a cross-linking approach is a good alternative to raise the compatibility of the PLA matrix with BSF. In addition, the production method using extrusion and injection moulding of BSF/PLA composite was found to have excellent mechanical properties. It was concluded that a prolonged analysis would help pave the way for an extended scope and prospect for the possible end-use of BSF reinforced PLA composite applications.

Bajpai et al. [102] studied the tribology of natural fibre-enhanced PLA composites and found that the incorporation of natural fibre sheets into the PLA matrix had significantly boosted the properties of neat polymer wear. In order to create a laminated composite using the hot compression process, natural fibres, for example, nettle, *grewia optiva*, and sisal were integrated with the PLA polymer. The operating parameters were set such that the loads applied were between 10 N and 30 N, the sliding speeds were between 1 m/s and 3 m/s, and the sliding distances were between 1000 m and 3000 m. The specimens were examined for friction and wear characteristics of the produced composites under dry surface conditions. The results of the experiment showed that there were decrements in the friction coefficient of 10%–44% relative to neat PLA and the real wear rate of composites produced by more than 70%. This research has established and demonstrated that natural fibres can be an efficient alternative to tribological material reinforcement implementations.

##### Bast

To date, several studies have investigated the reinforcement of natural fibre with PLA composite. In this section, reinforced PLA composite using natural fibres originated from non-wood basts, for example, kenaf, jute, hemp and flax are reviewed. Graupner et al. [103] investigated the mechanical characteristics of natural and artificial cellulose fibres reinforced with PLA composite. Various types of natural fibre composites, for example, hemp, cotton, kenaf and man-made cellulose fibres with different characteristics were manufactured by compression moulding with a fibre mass of 40% and the use of PLA. Kenaf and hemp/PLA composites showed promising results in their tensile strength and young modulus values, while cotton/PLA exhibited strong collision features. Meanwhile, lyocell/PLA composites displayed promising result in all three features. In a nutshell, the study revealed the distinct characteristics of the composites that are able to be used for various technical applications, each meeting unique standards.

A few studies have started to investigate the use of non-wood jute derived natural fibre polymer composite reinforcement with PLA [104,105]. The work of Jiang et al. [104] on jute/PLA composite hydrothermal ageing and structural damage found by X-ray tomography. The study claimed that biodegradable PLA composites reinforced with natural fibre could replace conventional composites reinforced with synthetic fibre, but they might experience hygrothermal ageing from the combined effects of heat and moisture. The effect of 50 °C liquid ageing on water absorption, chemical fibre, matrix degradation, and the tensile properties of the composite jute fibre/PLA were associated with internal structure modifications of the composite jute fibre/PLA observed on X-ray computed tomography. The result showed that the hygrothermal ageing caused the jute fibre/PLA composite to reduce in strength, tensile elastic modulus, ductility, and mechanical properties. In conclusion, X-ray tomography will determine the effects of hygrothermal degradation of a composite jute fibre/PLA.

A variety of definitions of the term PLA have been suggested. This paper will use the definition suggested by Yu et al. [105], who saw it as PLA is a linear aliphatic thermoplastic polyester manufactured with high stiffness and UV stability from renewable resources. The research reported that PLA is produced by two methods; ring-opening polymerisation of lactide and polycondensation of lactic acid, while the combination PLA/jute fibre material indicated that total tensile strength was slightly above that of the PLA. Interestingly, the PLA was observed to have the lowest tensile strength than PLA based composites. The results of this study revealed that with the incorporation of the fibre, the mechanical properties of materials dependent on PLA will increase and then decline until the fibre content is above 30%.

Oksman and Selin [106] discovered that plastics and composites from PLA showed that in a composite system, where natural fibres are used as reinforcements, PLA may be used as the matrix. Flax-reinforced PLA composites that can easily be extruded and compressed were found to be 50% stronger than many other thermoplastic composites reinforced with flax that are currently used in automotive panels. With the addition of 30% flax fibres, the PLA stiffness was increased from 3.4 GPa to 8.4 GPa. The results of this research indicated that the possibility of using typical production methods is an important element in the industrial use of renewable resources. There were no problems with extrusion and compression moulding in PLA/flax composites in this situation, and they can be easily processed.

In addition, an informative study by Omar et al. [107] involved the application of kenaf fibre reinforced composite in the automotive industry. The study reviewed the latest findings in kenaf fibre reinforced composite. In contrast to synthetic fibres, natural fibre materials in the automotive sector could possess several benefits, for example, weight and price reduction, recyclability, renewability and being environmentally friendly. The results suggested that kenaf fibres have fewer environmental impacts from the processing of natural fibres compared to the manufacturing of glass fibres, the higher natural fibre content in the composite replaces the matrix (synthetic polymer), higher fuel efficiency and lower phase emissions due to weight reduction, and energy and carbon offsets from end-of-life natural fibre incineration [108]. The research concluded that better kenaf plantation procedures are also needed to be practised upstream in order to guarantee a consistent availability of good kenaf fibres and to satisfy the growing demand for kenaf biocomposites.

In an investigation conducted by Zhang et al. [109] on flame retardancy of flax/PLA biocomposites, it was discovered that the bio-inspired approach to fibre surface modification on flax/PLA composites could enhance flame retardancy of the material. Raw flax fibre was first coated with a thin adhesive polydopamine (PDA) film in an aqueous dopamine solution followed by iron phosphonate growth on the fibre surface in situ. In order to prepare a flame retardant biocomposite, the altered flax fibre was added to PLA. The improved composite showed reductions of 16% and 21% in peak heat release rate and total smoke generation, respectively, with a limited amount of fibre surface flame retardant. In summary, this research revealed a novel approach to high-performing reinforced polymer composite preparation of flame retardant material.

For a comprehensive understanding, detailed research on flame retardancy of flax/PLA composites was performed by Pornwannachai et al. [110]. Their study on fire-resistant natural fibre reinforced flame retardant textiles discovered that by treating fabrics with common flame retardants, composites made from natural thermoplastic blended materials were often effectively flame retarded before melting. The melting of intermingled flax thermoplastic fibre was used to prepare thermoplastic composites. Before composite preparation with different common flame retardants (FRs) for textiles, fabrics were prepared. UL-94 flame output tests showed that only the organophosphate FR passed the test while the flax/PLA control and the rest of the flame-retardant composite specimens failed. On the opposite hand, the entire flame retarded flax/PLA samples reached a V0 rating which represented improved flame retardant properties of the composites. However, the study concluded that each fire retardant had also decreased the mechanical properties of the laminates, although giving the composite laminate high fire-retardant properties.

##### Seed/fruit

Previous research on natural fibres was focused on reinforcing natural fibre with PLA composite. In a study conducted by Jang et al. [111] on the flammability of reinforced PLA composites with coconut fibre, the mechanical and thermophysical behaviour of composite treated with plasma therapy was established. The coconut fibre interfacial adhesion strength was increased by the means of plasma treatment. In most cases, the coconut fibre/PLA will shrink in size with an increase in fibre weight after surface treatment using plasma. To be precise, the longitudinal direction of composites has higher shrinkage than the transverse direction. In this research, it was stated that plasma treatment could be important in optimizing the mechanical and thermophysical properties of natural fibre reinforced composites.

Graupner [10] proposed the usage of lignin as a promoter to natural adhesion in composites of cotton fibre-reinforced PLA. This research examined the method of lignin reinforcement with biodegradable thermoplastic cotton as a natural adhesion promoter that influenced the composites’ mechanical properties. Further data collection was required to determine whether compression moulding affected fibre mass proportions by 40%. The findings will be of interest to the composites’ fractured surfaces when examined using electron scanning microscopy (SEM). The findings of the composite studies revealed that cellulose affected the cotton/PLA composite characteristics. From SEM images, it was found that by adding lignin, the correlation between fibre and matrix will be strengthened. It was possible to enhance tensile characteristics by reducing the impact properties.

##### Grass

In this section, several studies that investigated the reinforcement of grass originated natural fibre with PLA composite will be discussed, which will include bamboo fibre [112,113], elephant grass [114], and switch grass. Previous research by Gunti et al. [114] on mechanical and degradation properties for PLA reinforced jute, sisal and elephant grass-based composites showed that the mechanical properties of elephant grass composite were superior compared to jute and sisal. The composites were synthesized using an injection moulding method with various percentages of untreated and treated fibres. Furthermore, the tensile strengths of the PLA composite with treated elephant grass were 18.14% and 24% higher at 20% fibre loading than that of the treated jute/PLA composite and plain PLA. This research showed that the integration of fibres into the PLA matrix had greatly increased the strength and strengthened the modulus.

A study by Sukmawan et al. [112] on strength assessment of bamboo fibre-reinforced cross-ply green composite laminates had revealed the impacts of the fibre material on the mechanical properties and failure characteristics. Laminate PLA/bamboo fibre cross-ply composite (0/90) was fabricated by using the hand-layup method before hot pressing using dispersion type biodegradable PLA. The outcome showed that the PLA/bamboo fibre composite was like that of conventional glass fibre reinforced laminate plastic and the basic strength was three times greater than mild steel. The research concluded that PLA/bamboo fibre laminate cross-ply (0/90) might be a substitute for glass fibre-reinforced composites with the potential to be used in sandwich structures as a skin material.

A study of bamboo fibre reinforced biocomposites was conducted by Abdul Khalil et al. [113]. In the last few years, the usage of bamboo fibres as insulation in composite materials has increased tremendously and has experienced a large development in response to the increasing need for the production of biodegradable and renewable materials. This result is somewhat counterintuitive; the rapid development in technology for producing goods makes it possible for customers to make a suitable choice and to have their own desirable preferences. This study has extended their quality skills through the use of raw materials such as bamboo fibre, which is stronger and can be used to produce sustainable high-end manufacturing products of high quality.

##### Straw fibre

Crop straw is a large-scale, low-price and sustainable farm waste. For the reuse of agricultural waste, research into straw fibre composite materials is very important. According to Elmessiry and Deeb [115], one of the difficulties of modern intensive farming is agricultural waste, which requires novel solutions for useful commercial use. The production of composites from sustainable raw materials has increased considerably over the last years as they are environmentally friendly materials. Thus, in recent years, many researchers had put more focused on straw fibre-reinforced composites including wheat straw [115,116], rice straw [117,118], corn straw [119], soy straw [120] and abutilon fibre [121].

A heated two roll-mill was used to prepare the poly (lactic acid)/rice straw (RS) composites by Mat Zubir et al. [117] using different ratios of RS. They investigated the mechanical performance of prepared PLA/RS composites. The tensile strength and elongation of the composite (E_b_) decreases with the rice straw fibre content increasing from 5% to 25% with the Young’s modulus increasing. These improvements also reported by Nyambo et al. [116]. They had discovered that enhanced tensile strength (20%) and flexural strength (14%) of the composites had increased considerably in line with that of the neat polymer compared to the 3 and 5 phr PLA-g-MA addition to composites. Plus, the strong interfacial adhesion between the fibre and matrix was due to the observed enhanced strength.

Pradhan et al. [120] had studied the compostability and biodegradation degree of soy straw/PLA and also wheat straw biocomposites. From their discoveries, it is found that PLA composites have shown to be clearly compostable materials for untreated soy and wheat straw. The deterioration of the PLA component is improved due to the existence of the natural biomass, indicating that modified/treated components can be used in composites. This discovery raises the chance of adopting a changed or handled biomass in the composites as any lack of deterioration that might occur due to the biomass alteration may be leveraged, due to the inclusion of readily degradable biomass components with the priming or favourable results.

On the other hand, Ding [119] had prepared corn straw fibre/PLA composite via hot pressing process, and its effect on the mechanical properties and degradation performance had been studied. The findings show that the mechanical properties of the composites first increased and then decreased with the rise of fibre content of the cornstalk (tensile strength and elongation during breakage). At 10%, the elongation ratio of break was 20.3%. The ratio of breakage is 10%. When the corn stem fibre content was 13%, the composite tensile strength hit 24.38 MPa. The intensity of corn straw fibre and rate of degradation of polylactic acid composites increased after 120 days of degradation. At same time, the mass loss of composites increased and the PLA molecular weight decreased more rapidly with the rise in corn straw fibre.

Abutilon fibre-reinforced PLA natural composites had been characterized and studied by Wang et al. [121]. This research uses a melting mix and an extruder to prepare biocomposites of PLA and abutilon fibres The DSC findings show that the fibres have acted as a central agent, resulting in an improvement in PLA crystallization. The findings also show that abutilon fibres have increased the thermal stability of PLA. In addition, higher storage modulus values due to heavy interfacial adhesion are observed. Thetan delta is also diminished by fibre material being added to the PLA matrix, which reduces the mobility of PLA polymer molecules in the presence of fibres. The enhancement of the properties and energy absorption of such biocomposites indicates that abutilon fibres have a tremendous potential as a refinement of green composites.

#### 5.2.3. Wood Natural Fibre/PLA Composite

A number of scientists have begun to examine the reinforcement of natural fibre with PLA composite. A study by Ozyhar et al. [122] on the effect of the functional mineral additive on the properties of the material and the processability of PLA reinforced wood fibre (WF) composites had analysed the use of alkenyl succinic anhydride (ASA) combined with calcium carbonate to act as the practical mineral supplement for WF reinforced PLA composites. With additional mineral amounts of 10, 20, and 30 wt.%, respectively, the effect of the number of minerals on the material properties of 40% fibre reinforcement PLA composites was investigated. The findings indicated that up to 20 wt.% of PLA can be substituted with ASA while preserving material properties. The research concluded that the addition of ASA-treated calcium carbonate had improved fibre adhesion with the PLA, enabling the composite formulation to substantially reduce the content of PLA while maintaining the properties of the material.

In a comprehensive study on PLA/natural fibre composites by Zhang et al. [123], the analysis was conducted as an essential first step towards the use of a mixture of modified natural fibres such as modified bamboo fibre, coconut fibre, and WF as reinforcing materials in the PLA matrix. Via the casting process, PLA composites with three sorts of natural fibres, like bamboo fibres, wood fibres, and coconut fibres were prepared. The results showed that adding three sorts of natural fibres could both strengthen the mechanical and thermal properties of composites, which the durability of composites might be further improved by natural fibres after modification. Additionally, as compared, the composite of the PLA/coconut fibre had the most excellent lastingness and thermal properties. This study presented a viable approach for the PLA industry to be introduced. In conclusion, this study offered a feasible method for the implementation of the PLA industry.

Du et al. [124] discovered that the inclusion of pulp fibres had improved the crystallisation of PLA and its tensile strength. Polymer composites were produced with natural fibres of PLA and cellulose, incorporating the process of shaping wet-layered fibre sheets with conventional composite manufacturing methods. The highest fibre amounts for maximum composite strength were 40% for high yield hardwood pulp fibre and 50% for high yield softwood and kraft pulp fibre. The highest tensile strength achieved was 121 MPa, almost double the natural PLA’s tensile strength. The results indicated that the inclusion of pulp fibres had successfully improved the composite storage modulus, elasticity, and even enhanced PLA crystallization. However, there were no major changes in the transition temperature of the composite glass and PLA crystallinity observed.

#### 5.2.4. Natural Mineral Fibre/PLA Biocomposites

One such material of interest currently compared to other natural fibres is natural mineral fibre, which is already being extensively used due to its cost effective, strong mechanical, physical as well as its chemical properties and biodegradable. Mineral fibres used as reinforcement material for composites including basalt [125] and asbestos fibre, via specific treatments. Recent research by Sang et al. [126] on the fabrication of PLA composites strengthened by short basalt fibre and their feasible assessment for 3D-printing applications evaluated the performance of KH550-treated composite reinforced PLA/basalt fibre (KBF) as a possible 3D-printed feedstock. PLA/KBF feedstock filaments were successfully manufactured and printed using the fused-deposition modelling (FDM) technique to variable shape and size pieces. The results indicated that PLA/KBF exhibited similar tensile properties and better flexural properties than PLA/carbon fibre (CF), which can be attributed to the highly complex PLA/CF viscosity which affected the interlayer adhesion. PLA/KBF was shown by the latest research as a technologically enhanced and cheap feedstock for uses in complex design and adjustable scale 3D-printing.

A study by Kurniawan et al. [127] was conducted on the effects of plasma polymerization on silane treated basalt fibre in order to evaluate the mechanical and thermal properties of the basalt fibre reinforced PLA composites. The findings revealed that the composite’s mechanical properties were enhanced 45% and 18% higher than the untreated properties respectively. This enhancement also related to the optimized time of 4.5 min irradiating the basalt fibres.

### 5.3. Processing Method Developments of Natural Fibre/PLA Composite

Natural fibre reinforcement with PLA composite has been the subject of a great deal of previous natural fibre studies. In a study conducted by Khan et al. [128], the mechanical properties of reinforced PLA composites for woven jute fabrics, an eco-friendly bio-composite was introduced as an alternative to the non-biodegradable synthetic fibre composite. The hot press moulding process was used to prepare the PLA reinforced plain-woven jute fabric (WJF). The average values of tensile strength, tensile modulus, flexural strength, flexural modulus, and impact strength of raw warp directed woven jute composite were increased by approximately 103%, 211%, 95.2%, 42.4%, and 85.9%, respectively, and the strain at maximum tensile stress was increased by 11.7% after the reinforcement. It was deduced that jute fabric composites based on PLA can be a suitable synthetic fibre composite alternative even for high load-bearing applications.

The value of natural fibre reinforced polymer composites has been illustrated by several studies. In an engaging study by Ogin et al. [89] on the components, design, and generic degradation of composite materials, the fundamental components of composite materials and the generic defects arising from the manufacturing process and the external loading of the material were described. Due to resin shrinkage, common process-related defects include porosity, shrinkage cracking, and fibre matrix debonding were observed. The curing kinetics, e.g., temperature cycles, gelation mechanisms, and resin shrinkage, resulted in internal curing and temperature stresses owing to the difference in thermal expansion coefficients (CTE) between the various basic components, which could lead to material defects too. This study also emphasised the essential component of material architecture optimization that was the avoidance of excess material use (weight reduction) and unnecessary strength, given that structural performance is only required in the parts and directions of the structure that bear stress.

Many studies have investigated natural fibres systematically reinforced PLA composites. There is a growing body of literature recognized by Bergeret and Benezet [129] about natural fibre-reinforced bio foams. Previous studies investigated their criteria for selection on starches and PLA as major bio-based and biodegradable polymers for biofoam applications. A number of techniques were developed such as blowing agent to melt extrusion to produce starch foams. The starch foam of natural fibres resulted in a decrease in density by up to 33% and void content of 48% for cellulose to increase mechanical properties of fibre-reinforced PLA foams.

Jauhari et al. [130] studied natural fibre reinforced composite laminates. By using this approach, researchers have been able to investigate natural fibre reinforcement by assembling the long or short bundles of natural fibres. In polymeric composite terminology, it created a flat sheet between one to tens layers of fibres. Different methods were proposed to classify the interlock of the fibres themselves with a binder in mechanical properties. To understand the conduct of FRPs under axial load, a PLA drop-off laminate was also modelled and analysed using ANSYS Software. The results of this investigation showed that the layers were held together to keep these materials together.

A study conducted by Lim et al. [73] on PLA processing technology, which could be considered as an option for mitigating waste management problem and reducing dependency on synthetic plastic for food packaging. This was attributed to the biodegradability and eco-friendly properties of PLA-based composite. It was stated that there are various PLA processing approaches which include methods of injection moulding, extrusion, and thermoforming fibre spinning. Demand for agricultural feedstock to produce PLA will also rise as the use of PLA continues to develop. Overall, the existing data highlighted the importance of the developments of sustainable natural sources in order to address possible alternative raw materials in the food supply chains.

In one study by Nechwatal et al. [131], the characterisation and use of natural fibre properties for composites were studied. The reinforcement of natural fibre had led to increasing composites’ tensile strength and Young’s modulus. Moreover, a new system for the processing of long fibre reinforced thermoplastic granules using regular plastic equipment was also proposed. In order to test a thread-like structure, the single fibre and fibre bundle tests were created. The fibre bundle test was known for its speed, whereas the precision of the single fibre test was established. The emphasis of this experiment was more on precision and hence, the single fibre test was used. A significant alternative to the current processes, for example, pultrusion or extrusion are the processes that involve long fibre granules. The new long fibre granules have a helical structure that can permit large fibre length for the composite.

Many studies have investigated natural fibres reinforced PLA composites in systematic ways. There is a growing body of literature recognized by Van De Velde and Kiekens [132] on thermoplastic pultrusion of natural fibre reinforced composites. As a reinforcement material for composites, natural flax fibre offered good opportunities in terms of mechanical characteristics and environmental advantage. One method to satisfy continuous demand for composites is pultrusion. As mentioned in the study, the capacity to become a major new force in the pultrusion industry was demonstrated by thermoplastic pultrusion and showed that the growth of thermoplastic pultruded composites reinforced with flax fibre might respond to the need for constantly reinforced profiles that are environmentally friendly.

**Table 2:** Reported work on natural fibre reinforced PLA composites.

Polymer	Natural Fibre	Effect of Reinforcement	Mechanical Strength			Reference
			Tensile (MPa)	Flexural (MPa)	Impact (kJ/m^2^)	
PLA	Bamboo fibre	-Increased mechanical properties -Bamboo fibre was the most effective in increasing tensile strength	54	-	-	[133]
PLA	Wood fibre	-Improved the fibre adhesion with the PLA,	73.8	-	21.5	[122]
PLA	Hard wood high yield pulp	-The incorporation of pulp fibres significantly increased the composite storage moduli and elasticity	90	-	-	[124]
PLA	Soft wood high yield pulp	-The incorporation of pulp fibres significantly increased the composite storage moduli and elasticity	110	-	-	[124]
PLA	Kraft	-Suggesting the fibre–fibre bond also positively contributed to the composites’ strengths	121	-	-	[124]
PLA	Wood fibre	-Improved mechanical and thermal properties of composites	23.0	-	-	[123]
PLA	Vetiver fibre	-Increased mechanical properties -Bamboo fibre was the most effective in increasing tensile strength	48	-	-	[133]
PLA	Coconut fibre	-Increased mechanical properties -Bamboo fibre was the most effective in increasing tensile strength	50	-	-	[133]
PLA	Kenaf fibre	-Comparable mechanical and physical properties to glass-fibre composite -Safe for environment	-	-	-	[107]
PLA	Jute fibre	-Elephant grass fibre had higher tensile strength and flexural strength than treated Jute/PLA composite and plain PLA -Untreated composite with elephant grass, jute, and sisal fibre had higher impact strength than plain PLA	65	112	5.3	[114]
PLA	Sisal fibre	-Elephant grass fibre had higher tensile strength and flexural strength than treated Jute/PLA composite and plain PLA -Untreated composite with elephant grass, jute, and sisal fibre had higher impact strength than plain PLA	62	110	5.3	[114]
PLA	Elephant grass	-Elephant grass fibre had higher tensile strength and flexural strength than treated Jute/PLA composite and plain PLA -Untreated composite with elephant grass, jute, and sisal fibre had higher impact strength than plain PLA	55	86	3.0	[114]
PLA	Flax fibre	-Showed high Limiting Oxygen Index (LOI) of 26.1% -Achieved V-2 rating	59.1	-	-	[109]
PLA	Wheat straw	-Excellent tensile mechanical properties	62	98	27	[116]
PLA	Rice straw fibre	-Increased the shear modulus and impact strength	50	-	-	[117]
PLA	Corn straw fibre	-Shorter curing time and enhanced mechanical properties.	24.38	-	-	[119]
PLA	Abutilon fibre	-Increased the shear modulus and impact strength	53	-	1.7	[121]
PLA	Woven jute fibre	-Increased mechanical properties	81	82	16.4	[128]
PLA	Hemp fibre	-Improved mechanical properties	54.6	112.7	-	[134]
PLA	Jute fibre	-Water absorption during ageing caused fibre/matrix bonding failure	60	-	-	[104]
PLA	Jute fibre	-Increased the shear modulus and impact strength	52	100	9	[105]
PLA	Ramie fibre	-Shorter curing time and enhanced mechanical properties.	48	105	10	[105]
PLA	Flax fibre	-Optimised mechanical properties of composites for automotive applications	53	-	12	[18]
PLA	Ramie fibre	-The composites had better mechanical properties than pure PLA	72	-	11	[135]
PLA	Hemp fibre	-Improved tensile, impact, and flexural properties	45	60	9.7	[136]
PLA	Hemp-lyocell fibre	-Improved tensile, impact, and flexural properties	60	102	21.7	[136]
PLA	Lyocell fibre	-Improved tensile, impact, and flexural properties	80	121	26	[136]
PLA	Flax fibre	-Flame retardant flax/PLA samples achieved V0 rating -All flame retardant reduced the mechanical properties	80	40	-	[110]
PLA	Basalt fibre	-PLA/KBF exhibited comparable tensile properties and superior flexural properties	79.5	128	5.6	[126]
PLA	Grewia optiva fibre	-Tensile strength of the composite increased by 75% of the neat polymer -Microwave joining was better than adhesive bonding	-	-	-	[137]
PLA	Palm fibre	-Increased mechanical strength	35	-	-	[138]
PLA	Kenaf fibre	-Increased mechanical strength	27	-	-	[138]
PLA	Manicaria Saccifera palm fibre	-Tensile strength, elastic modulus, and impact resistance were improved by 26%, 51% and 56%, respectively	68.45	133.12	26.62	[139]
PLA	Cordenka rayon fibres	--Mechanical properties improved	58	-	72	[140]
PLA	Kenaf fibre	-Very high tensile strength and Young’s modulus values	52.9	-	9	[103]
PLA	Jute fibres	-Increased tensile stiffness and strength significantly -Different improvements of the mechanical parameters	81.9	-	3	[141]
PLA	Keratin-based fibre	-Green and safe environment -Joints and bone fixtures to alleviate pain for patients	-	-	-	[142]
PLA	Flax fibre	-Mechanical properties improved -Tensile properties were in the same range	253.7	-	-	[143]
PLA	Flax fibre	-Tensile properties improved -Tensile strength and modulus of flax fibre/PLLA composite close to glass fibres	100	-	-	[144]
PLA	Ramie, flax and cotton fibres	-Difference intrinsic viscosity and melt flow index	-	-	-	[145]
PLA	Kenaf fibre	-Excellent tensile mechanical properties	52.88	-	8.97	[10]
PLA	Kenaf fibres	-Perfectly improved features of the polymers -Suitable for construction materials application	-	-	-	[146]
PLA	Hemp fibres	-Increased PLA transcrystallinity -Improved chemical bonding	-	-	-	[147]
PLA	Plant fibres	-Susceptibility to chemical degradation	-	-	-	[148]
PLA	Woven flax and jute fabrics	-Flax fibre resulted in composites with better mechanical strength than the woven jute fibre composites	-	-	-	[149]
PLA	Abaca fibre	-No weight loss observed for neat PLA and PLA/AA-abaca composite, meanwhile the PLA/untreated abaca composite showed ca. 10% weight loss	-	-	-	[150]
PLA	Banana/sisal fibre	-Mechanical properties were enhanced	80	125	48	[101]
PLA	Lyocell fibre	-Very high tensile strength and Young’s modulus values -Good impact characteristics	81.8	-	39.7	[103]
PLA	Abaca fibres	-Increased tensile stiffness and strength significantly -Different improvements of the mechanical parameters	74	-	5	[141]
PLA	Coir, sisal and jutes fibres	-Can be used to replace petroleum-based polymer -High specific strength	16.17	29.26	46.17	[151]
PLA	Coconut fibre	-Improved mechanical and thermal properties of composites	23.5	-	-	[123]
PLA	Bamboo fibre	-Improved mechanical and thermal properties of composites	22.5	-	-	[123]
PLA	Bamboo fibre	-Tensile strength was comparable with ordinary glass fibre-reinforced plastics laminate -Three times higher specific strength than mild steel	210	-	-	[112]
PLA	Coconut fibre	-Excellent tensile mechanical properties	65	-	-	[111]
PLA	Bamboo fibre	-Thermal conductivity of the natural composite was lower than that of synthetic composites	-	-	-	[152]
PLA	Cotton fibre	-Very high tensile strength and Young’s modulus values -Good impact characteristics	41.2	-	28.7	[103]
PLA	Cotton fibre	-Increased stiffness, tensile strength, elongation at break, and impact strength	41.20	-	28.71	[10]
PLA	Bamboo fibre, vetiver grass fibre and coconut fibre	-Impact of strength decreased	-	-	1.8	[153]

## 6. 3D and 4D Printings of PLA Biocomposite

Polylactic acid, a thermoplastic aliphatic polyester is the main nature-based raw material for 3D printing. It is a wholly biodegradable thermoplastic polymer synthesised from renewable raw materials [8,154]. Among the entire 3D printing materials, PLA is one of the most common feedstocks used for additive manufacturing. Ease of processing is a key advantage of PLA, being one of the easiest materials to print, despite tend to slightly shrink after the 3D printing process [155]. The main advantage of PLA compared to acrylonitrile butadiene styrene (ABS) material is it does not need a heated platform during printing, hence PLA can be printed at low temperatures between 190–230 °C [156]. PLA also does not require complex post-processing since it can be treated with acetone or sanded when necessary and the supports are usually very easily removable. Many manufacturers fabricated PLA filament to be used with 3D and 4D printing such as Prusament, Amolen, Polymaker PolyMax, Proto Pasta, MatterHackers, Fillamentum, Colorfabb, Sunlu, and Paramount 3D. One of the most prominent PLA filament manufacturers is an Austrian company WeforYou. In other side, the German company named Evonik focuses on PLA development for medical sector. Whereas the American company NaturaWorks is another large producer of biopolymers, and the company Corbion, based in the Netherlands, centralises the development of high-performance resins with PLA.

Since the material is suitable for interaction with foods, this material is used as a replacement for petroleum-based plastics for packaging application, especially in the food industry [157]. PLA can be utilized in 3D printing using the FDM (fused deposition modelling) technology, which manufactures parts through the extrusion of thermoplastic filaments, and PLA is one of the commonly used materials for this technology.

**Figure 6:**
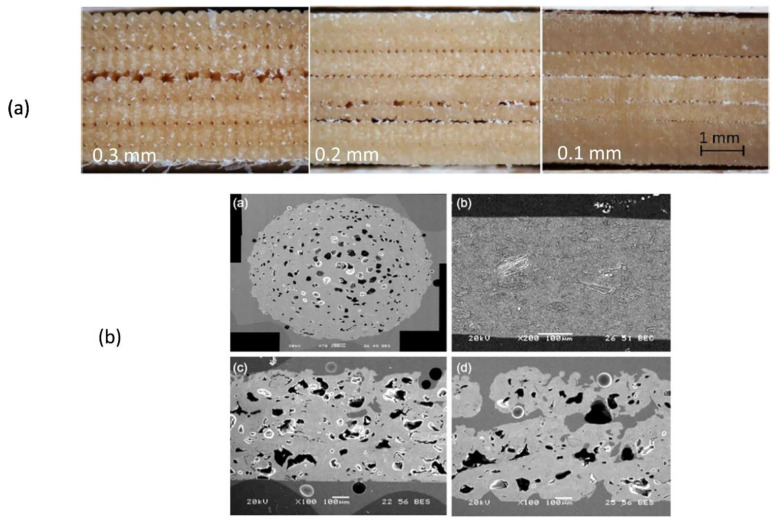
Short natural fibre reinforced PLA. (**a**) Effect of layer height on wood, 0.3, 0.2, and 0.1 mm [126]. Higher porosity content was observed with higher layer height. (**b**) Scanning electron micrograph of printed wood/PLA biocomposites with raw filament cross-section. Adapted from Le Duigou et al. [159] and Ayrilmis et al. [160] with copyright permission of Elsevier.

**Figure 7:**
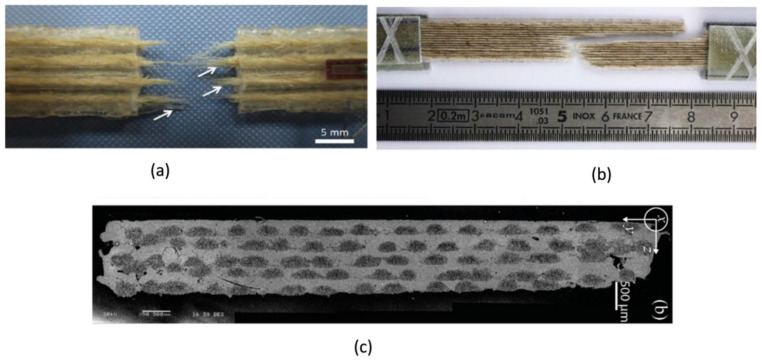
Continuous natural fibre (**a**) Fractured continuous unidirectional jute fibres/PLA sample printed with in-nozzle reinforced with various fibre debonding. (**b**) Fractured continuous unidirectional flax fibre/PLA sample printed with pre-impregnated filaments with a transverse crack followed by propagation along the tensile axis. (**c**) SEM micrograph of a cross-section of Flax/PLA composite microstructure. Adapted with copyright permission from Le Duigou et al. [143] and Matsuzaki et al. [161].

**Figure 8:**
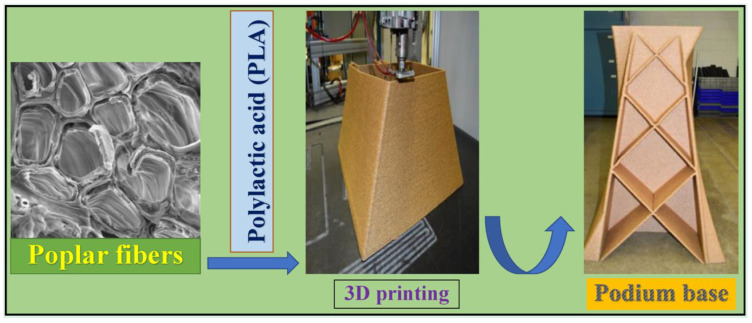
Large scale printing process with poplar/PLA composites. Adapted from Zhao X, Tekinalp H, Meng X, Ker D, Benson B, Pu Y, et al. [162]. ACS Appl Bio Mater 2019. Copyright (2019) American Chemical Society.

Composites are exceptionally advantageous in manufacturing lightweight with strong mechanical properties parts. The fibres function to contribute mechanical strength to parts without compromising weight, which is the primary factor of recognising composites as fibre reinforced materials [21,158]. There are two classes of reinforcements, short fibre (Figure 6) or continuous fibre (Figure 7). In short fibre case, chopped fibres consisting segments of less than a millimetre in length were mixed with thermoplastics such as PLA, ABS, or nylon into 3D printing plastics to raise the stiffness and to a lesser extent the strength of components. On the other hand, continuous addition of the fibres to the thermoplastics could be performed to produce stronger parts. Commonly, the most utilised fibre in 3D printing is carbon fibre along with glass, Kevlar or natural fibres. Figure 8 shows poplar as biofibre reinforcement in composites for large-scale 3D printing. Besides that, there are various hybrid materials that combine plastics with powders to give new colour, finish, or extra material properties. Usually for PLA, these materials are typically fabricated from 70% PLA biopolymer and another 30% hybrid natural material. For instance, wood-based filaments such as poplar, and non-wood filaments such as bamboo, jute, cork, wood dust kenaf, and so forth are combined with PLA to provide a more organic texture to the hybrid filament which in line with Sustainable Development Goals (SDGs) that are to (I) encourage inclusive and sustainable industrialisation and foster innovation, and (II) safeguard sustainable consumption and production patterns.

**Figure 9:**
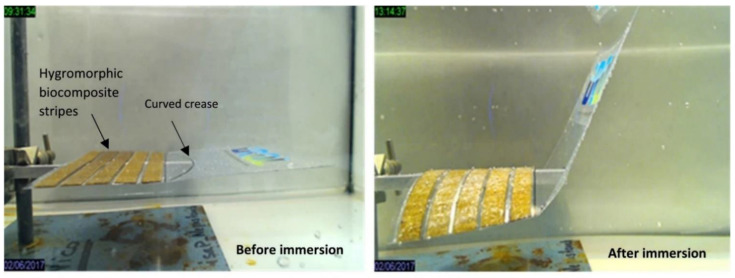
Moisture-induced deployable structure based on curved-line folding inspired by Aldrovanda, adapted from Le Duigou [175] with copyright permission of Elsevier.

**Figure 10:**
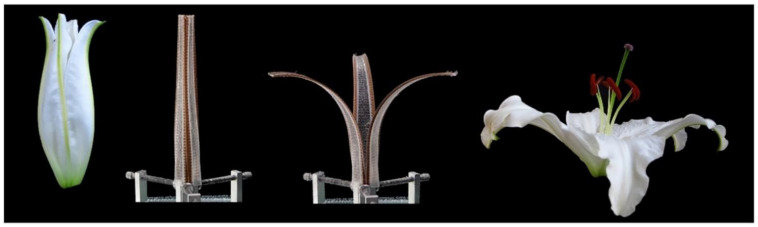
Edge growth causing a strain gradient in the Lilium ‘Casa Blanca’ drives the flower opening mechanism, a principle that is translated into a 4D printed mechanism with a wood-based hygromorph composite edge. Adapted from Poppinga et al. [176] with copyright permission of Oxford University Press.

**Figure 11:**
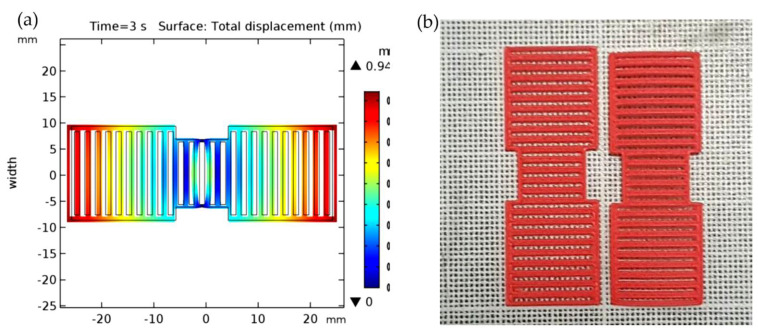
(**a**) PLA model deformation after 3 s. Uniformed vertical hollow spaces in 1 mm width. (**b**) Side by side comparison before and after adding stimulus. Left: a PLA model before adding stimulus and right: after adding the stimulus. Adapted from Alief et al. [177] with copyright permission of AIP Conference Proceedings.

Four dimensional printing is a relatively recent trend to develop 3D printed structures that can change their shape or properties over time [163,164,165]. The difference is that 4D printed objects can transform themselves over time, while 3D printed objects maintain fixed shape like any plastic or metal parts. The fourth dimension of 4D is the transformation over time, where 4D printing technology offers an output of smart structures by using new manufacturing techniques of 3D printing, advanced materials, and customized design. 4D printed objects need a stimulus to begin the deformation phase; the trigger can be an exposure to water, heat, light, or magnetic field [166,167,168,169]. Four dimensional printing technology is a combination of 3D printing, smart materials, and customized design for object transformation. The self-transformation of the structure is also called self-assembly as the structure can be designed to assemble itself. The concept of 4D printing is a smart structure that consists of rigid materials connected with expandable elements, or it can also be a whole structure made from expandable materials depending on what materials properties are needed and what are the applications [170,171]. The expandable elements can change their shape when exposed to certain stimuli, and this causes the hard parts to move or rotate, resulting in the whole structure transformation. The expandable element of smart materials can be hydrogel and elements with shape memory. A hydrogel is a polymeric material that is capable of absorbing a large amount of water [172,173]. It can be programmed to expand or shrink when there are changes in the external environmental conditions [163,174]. Hydrogels are biocompatible and easy to be modified. Elements with shape memory can be considered as smart materials due to their capability to return to their original shape from a deformed shape when stimuli are applied. Figure 9 shows the moisture-induced deployable structure based on curved-line folding inspired by Aldrovanda [175]. Hygromorph biocomposite (HBC) actuators made use of the transport properties of plant fibres to generate an out-of-plane displacement when a moisture gradient was present. Le Duigou et al. [175] developed a theoretical actuating response (curvature) formulation of maleic anhydride polypropylene (MAPP)/plant fibres (i.e., flax, jute, kenaf, and coir) based on bimetallic actuators theory (Figure 9). The result showed that the actuation was directly related to the fibres biochemical composition and microstructure, in which flax and jute fibres were observed and found to be the best candidates to be used in HBCs. Thus, PLA biopolymer might have huge potential to be used for hygromorph biocomposite (HBC) actuators. Simon Poppinga et al. [176] researched plant movements as concept generators for the development of biomimetic compliant mechanisms. Lilium ‘Casa Blanca’ or lily flower model was used in this experiment. The structure of the hygroscopic made up of wood-based hygromorph composite was translated into a 4D printed mechanism or printed under warm and dry conditions at a temperature of 21 °C and relative humidity of 18%. The composite then underwent deformation when submerged in water at the temperature of 19 °C. The submerged composite resulted to edge growth-driven actuation as identified from the petals of the lily flower (Figure 10). Their novel biomimetic compliant mechanisms highlighted the feasibility of modern printing techniques for designing and developing versatile tailored motion responses for technical applications. Alief et al. [177] conducted experiment on modelling the shape memory properties of 4D printed polylactic acid (PLA) for application of disk spacer in minimally invasive spinal fusion. Figure 11 shows the deformation of PLA model. They observed that specific PLA structure possessed thermal shape memory behaviour that can be thermo-mechanically trained into temporary shape and returned to its permanent shape when heated. Besides that, based on the simulation result, non-uniformed hollow spaces pattern displayed favourable result that could be a reference for future research in order to design the suitable pattern for the 4D PLA model. The possible advanced applications of 4D printing are medical devices for stents placed into blood vessels, drug capsules that release medicine, home appliances for control and that adjust according to humidity and heat, footwear and clothes, implants for humans and animals made from biocompatible materials, soft robots that can be activated without reliance on an electric device, smart valves and sensors for infrastructure lines [178,179,180,181,182,183].

## 7. Potential Applications of Natural Fibre Reinforced PLA Composite

Natural fibre reinforced PLA composite has a lot of potential usages. Possessing high mechanical strength similar to conventional glass fibre, it is seen as a potential substitution of glass fibre. Glass fibres are hard to biodegrade and is detrimental to the environment. Natural fibre reinforced PLA composite has high biodegradability and recyclability which is environmentally friendly. Among possible alternatives to the traditional fibres and synthetic polymers are PLA and natural fibres due to their renewability and ease of recycling [94]. As they have almost similar mechanical and physical properties, kenaf biocomposites have a promising ability to replace petrol-based composites such as glass-fibre composites [107] and used as packaging material. An increasing number of automotive parts and packaging materials using natural fibre composites is observed in India [184]. Thus, natural fibre reinforced PLA composite can even replace the conventional petroleum-based products with well-known environmental impacts.

Furthermore, due to the excellent mechanical strength of natural fibre reinforced PLA composites, they are favoured to be used in the automotive sector. A study reported that natural fibre reinforced PLA had comparable mechanical strength to glass fibre. The strength of the composites was comparable to that of normal laminated glass fibre-reinforced plastics, and the specific strength was 3 times higher than mild steel’s [112]. Jute fibre composite was found to have a higher damping behaviour, so jute fibre composites can be practical options as automotive parts since they possessed low vibration and noise [185]. Due to its relatively cheap cost, good properties, environmental friendliness and ease of manufacturing, natural fibre reinforced PLA composite can be applied in a wide range of applications, including the aerospace and automotive industries [100]. In the automotive industry, coconut fibre is used to make furniture in cars, while cotton is used for noise cancellation. Sometimes, wood fibre is also used as furniture and accessory in vehicles while flax, sisal, and hemp are applied in the refining of seatback linings and floor panels [96].

Besides the automotive industry, the construction sector also offers broad potential usages of natural fibre composite. The evaluation of building materials based on renewable resources such as natural fibres and their reinforcement in cement-based materials are being researched [98]. Structural beams and panels using bio-based composite materials were developed, manufactured and tested, particularly on plant oil-based resins along with natural fibre composite and composite building materials made from straw bales in the United States [184].

Natural fibre reinforced PLA composite has huge potential usage in the industry but its flammability might be a main concern. The flame retardant test on natural fibre reinforced PLA exhibited promising results and thus, this problem should not be worrying. The altered flax fibre reinforced PLA had a Limiting Oxygen Index (LOI) value of 26.1 with a UL-94 V-2 rating and the release of flammable gaseous during thermal decomposition of the PLA composite was suppressed after the addition of flame retardant, which enabled the modified composite to display high resistance to minor flammable sources of ignition [109]. The water absorption rate for all composites was observed to increase gently over the first 24 h before levelling off, and the rate of water absorption was increased in all composites as the fibre content was increased. The absorption rate was found to decrease with successive alkali treatment of the fibres [186].

## 8. Conclusions

The goal of this study is to investigate the potential of polylactic acid (PLA) composite reinforced with natural fibres to enhance the quality of the produced composite. The studies have proven that coconut and kenaf fibres exhibited fewer environmental effects from the processing of natural fibres, whereas elephant grass fibre reinforced PLA composite was stronger than the composites of jute and sisal. Essentially, the findings of the PLA matrix showed significant improvement in the smooth properties of polymer wear. Natural fibre reinforced PLA composites demonstrated their possibility to provide a possible backup to natural fabrics and polymers that are impossible and expensive to reuse. Numerous approaches have been employed to study the composites of natural fibre reinforced PLA, including flexural testing experiment, single-fibre test, fibre-bundle test, chemical treatments and plasma treatment. For example, tensile and flexural testing experiments on composites were created by strengthening a matrix of polyester resin lower than a new natural fibre. In the extrusion and compression moulding processes, PLA/flax composites did not demonstrate any difficulties and can be processed similarly to PP-based composites. In particular, for the mechanical properties of a composite, the quality of the fibre and matrix relationship is crucial. Fibre/matrix adhesion is a dynamic mechanism that deals with several variables. Generally, PLA natural fibre composites have durable mechanical properties. The beginning of 3D and 4D printings represents a great chance for PLA biocomposites to progress for the first time on the same time scale as their synthetic counterparts. Natural fibre reinforced PLA biocomposites have huge potential to be a new class of smart materials, namely hygromorph biocomposites, in which they are utilisable as raw materials in 4D printing to develop specialized shape-changing mechanisms and structures.

## Figures and Tables

**Table 1:** Chemical composition of selected common natural fibres.

Fibres	Holocellulose (wt%)		Lignin (wt%)	Ash (wt%)	Extractives (wt%)	Crystallinity (%)	Ref.
	Cellulose (wt%)	Hemicellulose (wt%)					
Sugar palm fibre	43.88	7.24	33.24	1.01	2.73	55.8	[46]
Wheat straw fibre	43.2 ± 0.15	34.1 ± 1.2	22.0 ± 3.1	-	-	57.5	[50]
Soy hull fibre	56.4 ± 0.92	12.5 ± 0.72	18.0 ± 2.5	-	-	59.8	[50]
Arecanut husk fibre	34.18	20.83	31.60	2.34	-	37	[51]
Helicteres isora plant	71 ± 2.6	3.1 ± 0.5	21 ± 0.9	-	-	38	[52]
Pineapple leaf fibre	81.27 ± 2.45	12.31 ± 1.35	3.46 ± 0.58	-	-	35.97	[53]
Ramie fibre	69.83	9.63	3.98	-	-	55.48	[54]
Oil palm mesocarp fibre (OPMF)	28.2 ± 0.8	32.7 ± 4.8	32.4 ± 4.0	-	6.5 ± 0.1	34.3	[55]
Oil palm empty fruit bunch (OPEFB)	37.1 ± 4.4	39.9 ± 0.75	18.6 ± 1.3	-	3.1 ± 3.4	45.0	[55]
Oil palm frond (OPF)	45.0 ± 0.6	32.0 ± 1.4	16.9 ± 0.4	-	2.3 ± 1.0	54.5	[55]
Oil palm empty fruit bunch (OPEFB) fibre	40 ± 2	23 ± 2	21 ± 1	-	2.0 ± 0.2	40	[56]
Rubber wood	45 ± 3	20 ± 2	29 ± 2	-	2.5 ± 0.5	46	[56]
Curauna fibre	70.2 ± 0.7	18.3 ± 0.8	9.3 ± 0.9	-	-	64	[57]
Banana fibre	7.5	74.9	7.9	0.01	9.6	15.0	[58]
Sugarcane bagasse	43.6	27.7	27.7	-	-	76	[59]
Kenaf bast	63.5 ± 0.5	17.6 ± 1.4	12.7 ± 1.5	2.2 ± 0.8	4.0 ± 1.0	48.2	[60]
Phoenix dactylifera palm leaflet	33.5	26.0	27.0	6.5	-	50	[61]
Phoenix dactylifera palm rachis	44.0	28.0	14.0	2.5	-	55	[61]
Kenaf core powder	80.26		23.58	-	-	48.1	[62]
Water hyacinth fibre	42.8	20.6	4.1	-	-	59.56	[63]
Wheat straw	43.2 ± 0.15	34.1 ± 1.2	22.0 ± 3.1	-	-	57.5	[64]
Sugar beet fibre	44.95 ± 0.09	25.40 ± 2.06	11.23 ± 1.66	17.67 ± 1.54	-	35.67	[65]
Mengkuang leaves	37.3 ± 0.6	34.4 ± 0.2	24 ± 0.8		2.5 ± 0.02	55.1	[66]

## Data Availability

No new data were created or analyzed in this study. Data sharing is not applicable to this article.
